# Evaluating the activity of temsirolimus in neuroendocrine cancer

**DOI:** 10.1038/sj.bjc.6603513

**Published:** 2006-12-12

**Authors:** P H O'Donnell, M J Ratain

**Affiliations:** 1 Section of Hematology/Oncology, Department of Medicine, University of Chicago, 5841 S Maryland Ave. MC 2115, Chicago, IL 60637, USA

**Sir**,

Duran *et al* (10 October 2006) reported the results of a phase II trial of temsirolimus in 37 patients with advanced neuroendocrine carcinomas (NEC). The authors cite an intent-to-treat response rate of 5.6% (two out of 36 patients who received temsiroliums achieved a partial response by RECIST criteria), and conclude therefore that ‘temsirolimus appears to have little activity and does not warrant further single-agent evaluation in advanced NEC’. Yet, Duran *et al* found that tumour control (partial response plus stable disease), measured as the percent tumour change from baseline, was achieved in 23 out of the 36 patients (63.9%) on temsirolimus. When it is considered that all patients in this trial had to have documented, progressive disease within 6 months of the study entry, the significant percentage of patients experiencing disease stabilisation on temsirolimus and the 1-year progression-free rate of 40.1% suggest drug activity beyond the natural course of the disease. Temsirolimus also compares favourably with other agents previously studied in NEC (Oberg, 2002). As a comparison, Faiss *et al* (2003) prospectively studied the use of interferon-alpha and the somatostatin analogue lanreotide in treatment-naïve NEC patients and found tumour control rates of 32% (lanreotide), 30% (interferon-alpha), and 25% (combination) at 1 year. Another study of 15 patients with metastatic NEC refractory to lanreotide examined the use of slow-release octreotide, and a partial response was seen in 7% of patients, stable disease in 40%, and progressive disease in 53% (Ricci *et al*, 2000). Duran *et al* also cite other recent reports of the use of sunitinib and, separately, gefitinib in NEC and acknowledge that the response rate and median time to progression with temsirolimus ‘compares favourably with other targeted therapies tested in this tumour population’.

This trial exemplifies the difficulty of interpreting the results of single-arm trials using the RECIST criteria (Michaelis and Ratain, 2006). This is especially true for noncytotoxic agents being studied in indolent diseases for which one would expect a high rate of stable disease in the absence of treatment. In fact, the response rate for temsirolimus in NEC compares favourably to that observed with sorafenib in advanced renal cell cancer, where the RECIST response rate is less than 5% (Ratain *et al*, 2006). Furthermore, if one compares the figures demonstrating ‘tumour size change from baseline’ for sorafenib in renal cell cancer to that of temsirolimus in NEC, the response distributions are nearly identical. However, the use of a randomised discontinuation design for the phase II trial of sorafenib demonstrated convincing evidence of activity and its subsequent widespread approval for the treatment of this disease. Rather than abandoning further evaluation of temsirolimus as a single-agent therapy for NEC, one should abandon the concept of single-arm studies of novel agents in neuroendocrine carcinomas.
